# Non-sequential protein structure alignment by conformational space annealing and local refinement

**DOI:** 10.1371/journal.pone.0210177

**Published:** 2019-01-30

**Authors:** InSuk Joung, Jong Yun Kim, Keehyoung Joo, Jooyoung Lee

**Affiliations:** 1 Center for In Silico Protein Science, Korea Institute for Advanced Study, Seoul, Korea; 2 School of Computational Sciences, Korea Institute for Advanced Study, Seoul, Korea; 3 Center for Advanced Computation, Korea Institute for Advanced Study, Seoul, Korea; Indian Institute of Science, INDIA

## Abstract

Protein structure alignment is an important tool for studying evolutionary biology and protein modeling. A tool which intensively searches for the globally optimal non-sequential alignments is rarely found. We propose ALIGN-CSA which shows improvement in scores, such as DALI-score, SP-score, SO-score and TM-score over the benchmark set including 286 cases. We performed benchmarking of existing popular alignment scoring functions, where the dependence of the search algorithm was effectively eliminated by using ALIGN-CSA. For the benchmarking, we set the minimum block size to 4 to prevent much fragmented alignments where the biological relevance of small alignment blocks is hard to interpret. With this condition, globally optimal alignments were searched by ALIGN-CSA using the four scoring functions listed above, and TM-score is found to be the most effective in generating alignments with longer match lengths and smaller RMSD values. However, DALI-score is the most effective in generating alignments similar to the manually curated reference alignments, which implies that DALI-score is more biologically relevant score. Due to the high demand on computational resources of ALIGN-CSA, we also propose a relatively fast local refinement method, which can control the minimum block size and whether to allow the reverse alignment. ALIGN-CSA can be used to obtain much improved alignment at the cost of relatively more extensive computation. For faster alignment, we propose a refinement protocol that improves the score of a given alignment obtained by various external tools. All programs are available from http://lee.kias.re.kr.

## Introduction

Because protein three-dimensional (3D) structures are closely related to their functions, protein structure alignment is an indispensable tool in investigating structural similarities of related proteins and classifying them in the evolution tree. The philosophy of the template-based modeling of protein 3D structures is based on the observation that proteins of similar amino acid sequences are of similar 3D structures. For this reason, protein structure alignment is frequently used not only for the 3D modeling of proteins when dealing with multiple templates but also for similarity evaluations of given structures.

A structure alignment of two given protein structures generally provides two types of information: the alignment score quantifying the similarity of the two structures and the alignment itself indicating the equivalence between aligned parts in terms of their C_*α*_ atom positions. In general, alignments are carried out on the residue basis, and aligned residues are not necessarily of identical side chains.

Over the past two decades, various protein structure alignment schemes have been proposed. Popular methods include DALI [1, 2], CE [3], ProSup [4], LGA [5], TMalign [6], and TopMatch [7]. In general, in the structure alignment of two given proteins, any C_*α*_ atom of one protein can be aligned to any C_*α*_ atom of the other protein. However, most of the alignment methods listed above allow only sequential alignment. That is, for any two aligned residue pairs, (*i*, *j*) and (*k*, *l*) with *i* < *k*, the condition of *j* < *l* is applied. This restriction reduces the number of possible alignments drastically, which in turn speeds up the processing time for dynamic programming [8] or combinatorial extension [3] algorithms during the sequential alignment. However, the topological orders of residues of two given proteins can be different from each other even when they share similar local structures. Therefore, topologically swapped local structures cannot be properly aligned within the sequential alignment scheme. Indeed, non-sequential alignments are found in not a few biological proteins [9]. Among the alignment methods listed above, only DALI allows non-sequential alignment. However, unfortunately, the non-sequential DALI alignment is not publicly available while only the sequential alignment version, DaliLite [10] is currently available.

Recently, many non-sequential alignments have been proposed including GANGSTA+ [11], FlexSnap [12], MICAN [13], CLICK [14] and SPalignNS [15]. Before we discuss the nature of the non-sequential alignment, we discuss an issue related to the alignment block size. An alignment block is defined as a part of the pairwise alignment between two proteins where all residues of the block are topologically consecutive in both proteins. Generally speaking, there is no restriction on the size of the alignment block. However, allowing small-sized alignment blocks tend to create much fragmented alignments, sometimes leading to complicated results with biologically questionable alignments. Concerns on this issue was discussed in an earlier publication [13]. To alleviate this problem, in DALI [2], the minimum block was set to to 4.

When the block size is greater than 1, whether the reverse alignment is allowed becomes another issue. Reverse alignments at first seem rather counter-intuitive in terms of the physico-chemical properties of the protein backbone chain that is known to contain a well-defined electric dipole moment. On the other hand, from the viewpoint of side-chains, it can be argued that the effect of protein backbone is less critical (e.g. when considering contacts of side-chain atoms with a ligand molecule). Because of this, the reverse alignment can be considered as a viable option.

The goal of the structure alignment is to obtain an optimal superposition between two given protein structures, where the match length (number of matched C_*α*_ atoms) is long and the root-mean-square deviation (RMSD) between the aligned residues is small. This is generally achieved by optimizing a scoring function which determines the quality of an alignment. Generally speaking, it is not straightforward to evaluate the efficiency of a score function *F*_1_ against another one *F*_2_, since often, *F*_1_ may produce a longer alignment with a poorer RMSD value while *F*_2_ generates a shorter alignment with a better RMSD value. However, if the alignment of *F*_1_ Pareto dominates that of *F*_2_, i.e., the alignment of *F*_1_ is superior in both the alignment length and the RMSD value to that of *F*_2_, one can argue that *F*_1_ is better than *F*_2_.

In spite of the conceptual simplicity, comparing scoring functions remains as a difficult task. This is because generating the globally optimal non-sequential alignment using a given score function is non-trivial and remains as a very difficult combinatorial optimization problem. Most alignment methods typically generate a few initial alignments in a heuristic manner and refine them to generate the best final alignment by optimizing a scoring function. Therefore, the quality of the final alignment depends on complicated factors: the quality of the initial alignments, the efficiency of the refinement algorithm, and the scoring function used. Although the novelty of various scoring functions has been claimed by many previous studies, the superiority of a scoring function independent from search algorithms has not been analyzed yet. In order to isolate the sole effect of the scoring function, the ability to perform efficient global optimization is indispensable.

In this research, we propose ALIGN-CSA by applying the conformational space annealing (CSA) [16] method to the non-sequential alignment problem. CSA has been successfully applied to various hard global optimization problems [16–23]. In many cases, CSA found more optimal solutions [20–22, 24–31] than found by conventional global optimization methods. CSA can be considered as a modified genetic algorithm. The key difference between CSA and conventional genetic algorithms lies in the way the diversity of the solution pool is controlled by employing a parameter called distance-cutoff, *D*_cut_. *D*_cut_ in CSA plays the role of temperature in simulated annealing and it controls the level of diversity in the solution pool. In the early stage of CSA, *D*_cut_ is kept large, so that solutions in the pool are quite diverse. As the value of *D*_cut_ decreases, the solutions begin to settle into various local minima, and their chances to be updated by more optimized solutions increase because their information is shared among them to perform crossover operations, which, in turn, can accelerate finding more optimal solutions.

The global search is especially useful when a high quality alignment is required but not adequate for aligning a protein to numerous proteins in a large database because of long runtime. As an alternative, we propose a refinement algorithm which can refine a given alignment using a chosen scoring function. The algorithm is inspired by the asymmetric greedy search (AGS) algorithm [15] and deep greedy switching (DGS) algorithm [32]. The refinement process may restrain the minimum alignment block size and control whether to allow the reverse alignment while greatly improving the score of the chosen scoring function.

We note that the current implementation of CSA for global search and the refinement protocol can deal any scoring function for alignment. In this work, we considered DALI-score [2], SP-score [33], SO-score [14], and TM-score [34] as defined below. For two given proteins *A* and *B* to be aligned, DALI-score is defined as follows:

$$\begin{array}{c} \text{DALI}=\sum_{i}^{n}\sum_{j}^{n}\{\begin{array}{cc} \left(0.2-\frac{\left|d_{ij}^{A}-d_{ij}^{B}\right|}{(d_{ij}^{A}+d_{ij}^{B})/2})\exp(\frac{-{\left|d_{ij}^{A}-d_{ij}^{B}\right|}^{2}}{d_{0,\text{DALI}}^{2}}\right) & i\neq j\\ 0.2 & i=j \end{array}. \end{array}$$ (1)

*n* is the number of aligned residue pairs and *d*_0,DALI_ = 20 Å. The distance matrix $d_{ij}^{A}$ contains the C_*α*_ − C_*α*_ distance of protein *A* between two corresponding residues from two aligned residue pairs *i* and *j*. So is $d_{ij}^{B}$ for protein *B*.

SP-score is defined as follows:

$$\begin{array}{c} \text{SP}=\frac{1}{3L^{1-\alpha }}\sum_{d_{ij}<2d_{0,\text{SP}}}^{n}(\frac{1}{1+{\left(d_{ij}/d_{0,\text{SP}}\right)}^{2}}-0.2). \end{array}$$ (2)

Core residues are defined as the residues where *d*_*ij*_ ≤ 2*d*_0,SP_ and neighboring residues are non-core residues located within 3*d*_0,SP_ of any core residue. *L* is defined as the sum of the following two numbers: the number of core residues and the average number of neighboring residues of all the core residues. *α* = 0.3 and *d*_0,SP_ = 4.0 Å.

SO-score, or the structure overlap score is defined as follows:

$$\begin{array}{c} \text{SO}=\frac{1}{\min(L_{A},L_{B})}\sum_{d_{ij}\leq d_{0,\text{SO}}}^{n}1, \end{array}$$ (3)

where *L*_*A*_ and *L*_*B*_ are the chain length of protein *A* and *B*, respectively and *d*_0,SO_ = 3.5 Å. We note that direct optimization of SO-score is technically problematic since the gradient of the formula is zero. Discrete conformational space are typically optimized by Monte Carlo quenching process. Without the gradient of the scoring function, local optimization cannot be performed efficiently. For this reason, we used a modified SO-score using the logistic function as follows:

$$\begin{array}{c} {\text{SO}}_{\text{L}}=\frac{1}{\min(L_{A},L_{B})}\sum_{ij}^{n}[1-\frac{1}{1+\exp(-k(d_{ij}-d_{0,\text{SO}}))}], \end{array}$$ (4)

where *k* = 10 Å^−1^. For both global and local alignments, we used SO_L_-score throughout the calculation, but the final scores were re-evaluated using the original definition of Eq 3. Actually, the values of the modified and original SO score make little difference.

TM-score is defined as follows:

$$\begin{array}{c} \text{TM}=\frac{1}{L_{N}}\sum_{ij}^{n}(\frac{1}{1+{\left(d_{ij}/d_{0,\text{TM}}\right)}^{2}}), \end{array}$$ (5)

where *L*_*N*_ is either *L*_*A*_ or *L*_*B*_ and $d_{0,\text{TM}}=1.24\sqrt[{3}]{L_{N}-15}-1.8$. TM-score always has two values which are normalized by either *L*_*A*_ or *L*_*B*_. In this research, we used the average value of the two as the objective scoring function for optimization.

## Methods

### Global alignment

To search globally optimal alignments, we used the CSA algorithm and it was implemented using pycsa [35]. Details of CSA are available elsewhere [16, 18], and, here, we provide a brief description of CSA and its implementation details applied for the study of protein structure alignment.

To apply CSA to an optimization problem, three ingredients should be provided: (1) a distance metric to measure the difference between two given solutions, (2) a local optimizer to improve a given solution, and (3) ways to generate daughter solutions from two parent solutions. The three prerequisites and details about the procedure are explained in S1 File.

### Refining alignments

We have devised a refinement algorithm to further improve the score of the final alignment of ALIGN-CSA. The refinement algorithm is a modified version of the AGS (Asymmetric Greedy Search) algorithm [15, 32] that was applied to the protein alignment problem in SPalignNS. The original version of AGS does not include any constraints on the requirement of minimum block size or whether to allow the reverse alignment. Another key difference between the current refinement algorithm and AGS is that in AGS only swapping moves were considered while we considered all possible deletion and addition of an alignment pair. This difference led to about 10–100 fold increase of accepted move operations in the current refinement procedure compared to AGS. It should be noted that for each move considered in this study, RMS fitting was performed to calculate the score of the move for SP, SO and TM.

In the current refinement procedure, by examining a given alignment to refine, we considered all possible local deletion and addition moves as described above, and all score-improving moves were stored in a move list together with their involved residue indices and the score differences. Then, the best score-improving move was taken, and this could invalidate some of the score-improving moves in the list because they were involved with the same residue. Therefore, they were removed from the list. From the beginning of the second iteration, among all the possible moves, only the moves involved with any residue affected by the previously accepted move were considered. If they improved the score, they were added into the list. The best-score improving move in the list was taken and the score improvement was re-calculated. If the move still improved the score, it was accepted. Otherwise, the move was discarded and the next best move was tried in the same way. As far as a move in the list was accepted, it proceeded to the next iteration to add moves in the list. This procedure was repeated until the the move list became empty.

When constraints on the requirement of minimum block size or whether to allow the reverse alignment were applied, we performed the following preprocessing on the given input alignment. When reverse alignment was forbidden, all violated blocks were removed. This may leave the input alignment as a null set, and if this happened, we kept only one aligned pair per each block positioned at the center. When the minimum block size was violated, the violated block was removed. If this led to a null set, we extended all the largest-block-size blocks of the input alignemnt to minimum-block-size blocks.

### Alignment datasets

In order to benchmark the efficiency of the methods proposed in this work, we used four alignment datasets. The first set, DALISET [2] contains 5 pairs of protein structures: 1lyzA/2lzmA, 1colA/1sdhA, 1acxA/1cobB, 1acxA/1tnfA, and 1acxA/1madH. DALISET is the reference set used for DALI-score in this study, and this set is the only set where the results of the original DALI method are available. The other three sets are taken from the benchmark sets used in SPalignNS [15] and CLICK [14]: a subset of HOMSTRAD [36] containing 64 pairs of difficult cases, the so-called “similar structure but different topology” (SSDT) set containing 199 pairs that includes many swapped domains and different topologies, and a subset of the RIPC [37] set containing 23 pairs.

## Results and discussion

### Performance comparison of global alignment between DALI-CSA and DALI

We applied CSA with DALI-score to DALISET. Because neither web-based service nor the source code of the original DALI method is currently available, direct comparison between ALIGN-CSA with DALI-score (DALI-CSA) and the original DALI method is possible only for these five pairs. It should be noted that DaliLite [10] and DALIX [38] perform only sequential alignments although they include ‘DALI’ in their method names. Therefore, our method is not compared with them in this research.

For each of five protein pairs of DALISET, ten independent ALIGN-CSA runs were carried out and each run produced 100 final alignments. For each of the fifty ALIGN-CSA runs of the five protein pairs, the solution by ALIGN-CSA was more optimal than the one in the reference [1] in terms of its DALI-score. Except 1acxA/1madH, all 10 runs of each pair produced identical optimal solutions suggesting that the obtained highest DALI-score alignment could be the globally optimal solution. For 1acxA/1madH, 6 out of 10 runs produced the identical highest DALI-score alignment, while the other 4 runs produced sub-optimal solutions (but still more optimal than the original DALI alignment). The results of ALIGN-CSA are summarised in Table 1 and the alignments are in Table A in S1 File.

**Table 1:** Comparison between the DALI-CSA alignment and the original DALI alignment is shown.

PDB IDs	DALI-score		Match Length		RMSD (Å)	
	DALI-CSA	Ref	DALI-CSA	Ref	DALI-CSA	Ref
1lyzA/2lzmA	258.69	256.32	86	86	4.59	4.37
1colA/1sdhA	474.77	471.73	118	118	3.49	3.48
1acxA/1cobB	397.80	376.51	95	90	2.97	2.94
1acxA/1tnfA	279.42	269.05	82	80	3.56	3.57
1acxA/1madH	322.99	246.79	84	74	2.75	3.71
Average	346.73	324.08	93.0	89.6	3.47	3.61

In CSA, the *D*_cut_ reduction speed determines the annealing speed. Fast reduction of *D*_cut_ generally accelerates the convergence of solutions but with the increased possibility of failure to search an important part of the solution space. The current setting for the *D*_cut_ reduction (see CSA Procedure) was chosen to balance the computational cost of CSA and the robustness of the solution. It should be noted that no higher DALI-score solutions than shown in Table 1 were obtained even when using slower annealing schedules than the current setting, which strongly implies that the DALI-CSA solutions correspond to the globally optimal DALI alignment solutions.

DALI-CSA found not only tentative global optimal alignments but also many additional sub-optimal alignments whose DALI-scores were still higher than the reference scores. The numbers of the alignments with higher DALI-scores were 2 (1lyzA/2lzmA), 3 (1colA/1sdhA), 19 (1acxA/1cobB), 39 (1acxA/1tnfA) and 279 (1acxA/1madH). These numbers were counted at the end of DALI-CSA runs, hence the actual number of alignments with higher DALI-scores than the reference value can be even greater than the number listed here. We observe that, on average, the DALI-CSA alignment is of longer match length and of smaller C_*α*_-RMSD.

### Effects of minimum block size and reverse/non-reverse alignment of DALI-CSA

In the original DALI method, all alignment blocks were constrained to be at least 4 residues long and reverse alignment was allowed. To understand the effect of these constraints and reverse alignment, we performed DALI-CSA using various minimum block sizes ranging from 2 to 8, and allowing and disallowing reverse alignments. In addition, alignment without any constraints, i.e., general non-sequential alignment was also investigated.

The effects of the constraint conditions of the DALI-CSA alignment are summarized in Tables 2–4. As the constraint condition becomes less strict, we observe that the DALI-score improves, the number of aligned residues tends to increase, and the RMSD value tend to decrease. That is, both the coverage and the accuracy of alignment increase as the constraint condition becomes less strict. The general non-sequential alignment result is summarised under the column of minimum block size 1.

**Table 2:** Match lengthes of various constraint conditions.

Block Size	8	7	6	5	4	3	2	1
PDB IDs	reverse allowed							
1lyzA/2lzmA	86	86	84	89	86	94	101	104
1colA/1sdhA	118	119	118	118	118	118	125	127
1acxA/1cobB	80	84	93	95	95	95	96	97
1acxA/1tnfA	78	78	76	82	82	87	89	88
1acxA/1madH	82	81	85	81	84	88	93	96
PDB IDs	reverse not allowed							
1lyzA/2lzmA	73	80	84	89	83	84	81	104
1colA/1sdhA	118	119	118	118	118	118	122	127
1acxA/1cobB	80	84	82	87	90	90	94	97
1acxA/1tnfA	79	78	76	76	77	80	85	88
1acxA/1madH	71	75	73	75	75	86	84	96

**Table 3:** RMSD (Å) of various constraint conditions.

Block Size	8	7	6	5	4	3	2	1
PDB IDs	reverse allowed							
1lyzA/2lzmA	5.03	4.75	4.61	4.21	4.59	4.64	12.08(3.32)	12.16(3.19)
1colA/1sdhA	3.58	3.60	3.56	3.48	3.49	3.46	3.17	2.69
1acxA/1cobB	2.99	3.03	3.25	3.16	2.97	2.81	2.65	2.56
1acxA/1tnfA	4.21	3.94	3.75	3.50	3.56	3.78	3.21	3.15
1acxA/1madH	3.42	3.29	3.38	3.01	2.75	3.35	2.79	2.65
PDB IDs	reverse not allowed							
1lyzA/2lzmA	4.64	4.39	4.65	4.75	4.06	3.85	3.17	12.16(3.19)
1colA/1sdhA	3.58	3.60	3.56	3.48	3.49	3.46	3.37	2.69
1acxA/1cobB	3.16	3.47	3.41	3.55	3.44	3.26	2.88	2.56
1acxA/1tnfA	4.35	4.05	3.75	3.75	4.01	3.77	3.88	3.15
1acxA/1madH	3.91	3.32	2.96	3.01	3.06	3.41	3.56	2.65

**Table 4:** DALI-score of various constraint conditions.

Block Size	8	7	6	5	4	3	2	1
PDB IDs	reverse allowed							
1lyzA/2lzmA	204.69	226.31	237.30	246.60	258.69	274.08	324.37	362.78
1colA/1sdhA	465.87	465.99	468.44	472.12	474.77	481.24	527.32	598.93
1acxA/1cobB	332.09	345.73	360.98	386.12	397.80	411.86	440.34	458.41
1acxA/1tnfA	227.90	248.36	255.53	259.75	279.42	300.88	318.34	336.31
1acxA/1madH	269.16	278.63	291.98	303.67	322.99	335.38	375.34	402.68
PDB IDs	reverse not allowed							
1lyzA/2lzmA	177.67	200.56	214.12	229.45	236.49	250.52	272.69	362.78
1colA/1sdhA	465.87	465.99	468.44	472.12	474.77	481.24	496.12	598.93
1acxA/1cobB	316.42	328.45	335.29	341.63	353.94	361.26	394.94	458.41
1acxA/1tnfA	220.19	241.52	255.53	255.53	259.76	273.07	291.56	336.31
1acxA/1madH	238.59	251.69	269.27	272.96	277.70	294.27	317.67	402.68

Another thing we noticed is that, as the constraint condition became less strict, the DALI-CSA result became less robust. For example, for the general non-sequential alignment with no minimum block size constraint, except 1acxA/1cobB, the best score alignment was reproduced with less frequency (see Table 5). Interestingly, the reproducibility also drops sometimes when the minimum block size gets higher. Although there is an exception such as 1acxA/1madH, generally the reproducibility looks rather high when the minimum block size is 4. It implies that the number of competing alignments is least with the minimum block size. To measure the quantitative difference among various best alignments separately obtained from individual runs, for each protein pair, the structure of the second protein was fit to that of the first protein using the given DALI alignment and then the relative C_*α*_-RMSD between two structures of the second protein was measured. In two cases, thus-measured largest RMSD value among the best alignments for general non-sequential alignment were fairly small (0.37 Å for 1atzA/1auoA and 0.22 Å for 1colA/1sdhA), suggesting that the variation among best alignments is rather small. But in the other two cases, the largest RMSD values were rather large (32.2 Å for 1lyzA/2lzmA and 38.4 Å for 1acxA/1madH), demonstrating that the variation among best alignments is significantly large. For these two cases, the generated DALI-CSA solutions with the general non-sequential alignment condition are quite different from each other and the results shown in Tables 2–4 might not correspond to the globally optimal solution, for which much more extensive computational resources will be required to obtain more robust results.

**Table 5:** Reproducibility of the best results. The number of DALI-CSA runs producing the same highest value and the total number of runs are shown. Each run collected 200 alignments.

Block Size	8	7	6	5	4	3	2	1
PDB IDs	reverse allowed							
1lyzA/2lzmA	1/20	5/5	5/5	5/5	5/5	9/10	1/40	1/40
1colA/1sdhA	5/5	2/5	5/5	5/5	5/5	4/10	1/40	3/30
1acxA/1cobB	5/5	5/5	5/5	5/5	5/5	10/10	20/20	20/20
1acxA/1tnfA	5/5	5/5	2/5	5/5	5/5	8/10	17/20	19/20
1acxA/1madH	4/10	4/10	2/20	4/5	2/5	6/10	9/20	4/20
PDB IDs	reverse not allowed							
1lyzA/2lzmA	5/5	2/5	5/5	5/5	5/5	5/5	16/20	-
1colA/1sdhA	5/5	4/5	5/5	5/5	5/5	5/5	9/20	-
1acxA/1cobB	5/5	5/5	5/5	5/5	5/5	5/5	19/20	-
1acxA/1tnfA	5/5	5/5	3/5	5/5	5/5	5/5	20/20	-
1acxA/1madH	5/5	2/5	4/5	2/10	5/5	5/5	5/20	-

**Fig 1:**
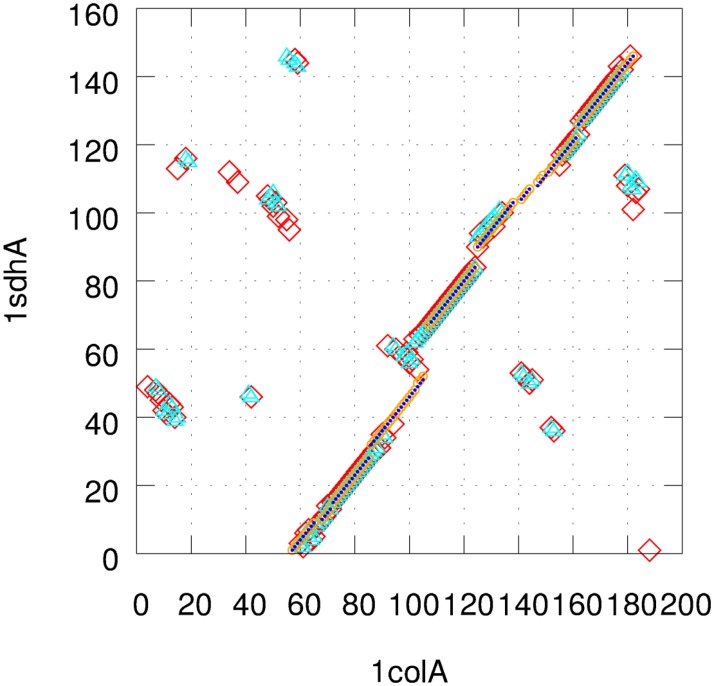
The effect of minimum block size is examined for 1colA/1sdhA using DALI-CSA. The best alignments using minimum block sizes of 1, 2, 3, and 4 are shown (1: red diamond, 2: cyan triangle, 3: orange circle, 4: blue dot). Reverse alignment is allowed in all cases.

It is interesting to observe the large RMSD values of 1lyzA/2lzmA in Table 3 for minimum block size of 1 and 2 with the allowed reverse alignment. By visual inspection of the alignment of 1lyzA/2lzmA, we found that one protein is aligned to the mirror image of the other protein resulting in the large RMSD values. The RMSD values measured with the corresponding mirror images are much reduced to 3.32 and 3.19 Å as shown in parentheses in Table 3. This problem originates from the fact that DALI is based on the distance matrix, for which the chirality of the protein chain molecule is missing. In addition to this problem, much fragmented alignment blocks are observed when using small minimum block sizes (see Fig 1). The biological relevance to these fragmented alignments is not clear and it makes hard to draw meaningful implication of non-sequential alignments in this case. The block size issue will be discussed again later.

### Comparison between ALIGN-CSA and external tools

All the scoring functions were optimized using the ALIGN-CSA algorithm for HOMSTRAD, SSDT, and RIPC sets. We used the four scoring functions, DALI, SO, SP, and TM for optimization. A single CSA run was carried out for each pair of proteins and collected 400 alignments. The minimum block size was restricted to 4 and reverse alignment was allowed. Three external tools were tested: CLICK, SPalignNS, and MICAN. When running the external tools, we used the default parameters of the tools but reverse alignment was allowed in running MICAN. Other tools allow reverse alignment by default. Essentially, it can be presumed that there is no minimum block size restriction on those tools. Table 6 shows the average alignment score, match length and RMSD. The table shows only the best average score for each score function. The highest DALI-score was obtained by MICAN and the highest SO-score was obtained by SPalignNS consistently. The alignment scores of CSA runs were always higher than those of the external tools. Since the external tools are not designed as global optimization tools, the results are not surprising. However, it is interesting that more optimized score obtained by CSA resulted in longer match lengths and larger RMSD values. This tendency was observed for all the test sets and score functions consistently.

**Table 6:** Comparisons of the average scores by ALIGN-CSA with the average scores by the best external tools out of SPalignNS, CLICK, and MICAN. In each CSA run, the minimum block size was 4 and the CSA run continued until a total of 400 alignments were generated.

Test set	Score	Best tool	Score			Match length		RMSD	
			Tool	CSA	*p*-value	Tool	CSA	Tool	CSA
HOMSTRAD	DALI	MICAN	433.3	488.5	<0.01	82.47	86.78	2.905	3.377
	SO	SPalignNS	0.7139	0.7319	0.05	72.28	86.45	1.999	3.764
	SP	SPalignNS	0.5628	0.5757	0.08	72.28	83.44	1.999	2.624
	TM	SPalignNS	0.5083	0.5387	<0.01	72.28	89.02	1.999	3.515
SSDT	DALI	MICAN	343.4	404.8	<0.01	79.57	85.37	2.975	3.417
	SO	SPalignNS	0.7160	0.7598	<0.01	71.33	88.62	1.917	3.906
	SP	CLICK	0.5194	0.5511	<0.01	77.21	83.63	2.251	2.751
	TM	CLICK	0.4954	0.5360	<0.01	77.21	92.84	2.251	3.642
RIPC	DALI	MICAN	910.7	1104.1	<0.01	141.48	158.78	2.93	4.422
	SO	SPalignNS	0.6538	0.6605	0.37	124.74	158.35	1.805	5.645
	SP	SPalignNS	0.6451	0.6771	0.01	124.74	136.35	1.805	2.491
	TM	MICAN	0.5405	0.5947	<0.01	141.48	174.22	2.93	4.478

If a scoring function is absolutely superior to the others, it is desirable to optimize alignments more by applying a global optimization method. However, showing the superiority of a scoring function is complicated. The alignments produced by the external tools are the results of the alignment algorithm as well as the scoring function they use. By using a global alignment tool like ALIGN-CSA, one can compare the effect of the scoring function on the alignments without the alignment algorithm being affected. The mathematical improvement of the score itself by ALIGN-CSA is obvious as shown in Table 6. Nonetheless, with the score functions tested in this article, unfortunately we could not find much merit in using the global alignment method if the purpose of the alignment is obtaining more biologically relevant alignment. Instead, one can use refinement algorithm for such a purpose, which will be discussed below. However, we still see a possibility that global alignment can be used in improving alignments if the scoring function is designed well (Fig C and Table B in S1 File).

### Performance of refinement process

**Fig 2:**
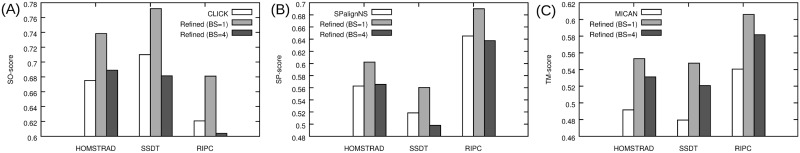
Scores of refined alignments. The alignments of CLICK, SPalignNS, and MICAN were refined by optimizing SO-score, SP-score and TM-score respectively. The block size were restricted to either 1 (BS = 1) or 4 (BS = 4). The bars show the average scores of the alignments measured from the three test sets.

The outputs of SPalignNS, CLICK and MICAN from the three benchmark sets, HOMSTRAD, SSDT and RIPC were further refined by the refinement algorithm proposed in this work. SPalignNS is clearly designed to maximize SP-score. However, the objective scoring functions for CLICK and MICAN are not clear. Nevertheless, CLICK is intended to maximize SO-score and MICAN is intended to maximize modified TM-score according to the references [13, 39]. Note that modified TM-score is slightly different from the original TM-score. We refined outputs of SPalignNS, CLICK and MICAN using the four score functions. In the refinement, reverse alignment was allowed and the block size was restrained to either 1 or 4. With the block size of 1, Fig 2 shows parts of refinement results, where CLICK is refined with SO-score, SPalignNS is refined with SP-score, and MICAN is refined with TM-score. We chose the specific combination because the pair of the alignment program and the scoring function is relevant. In other combinations of the program and the scoring function, the tendency is not different. All the other results are provided in Fig D in S1 File. The refinement algorithm clearly improves the score on average (several in the second significant digit, *p*-value < 0.01), which demonstrates the performance of the refinement process. The external tools arguably does not restrain the minimum block size. If the minimum block size greater than one is chosen in the refinement process, the decrement of the score is inevitable due to the more restrictive alignment condition. However, even with the minimum block size of 4, the scores of the refined alignments are similar to those of inputs. The result implies that the refined alignments are less fragmentary while the alignment score is more or less similar.

### Quality of input alignments for refinement

Table 7 summarizes all the refinement results. Here, the minimum block size was set to 4 in all cases. The input alignment to refine was taken from the method indicated by the first column. Unlike other alignment tools, TMalign is a sequential alignment tool. For each benchmark set, the results were generated using the outputs of the external tools as the input alignments.

**Table 7:** The average scores measured after refinement. The output alignments of the method indicated in the first column were refined using the four scoring functions indicated in the first row of the table as the objective function. The minimum block size was set to 4 and three benchmark sets (HOMSTRAD, SSDT, and RIPC) were tested. For each refined score function, the highest values are shown in bold face.

Score function	DALI	SP	SO	TM
HOMSTRAD				
CLICK	468.1	0.5604	0.6890	0.5242
SPalignNS	476.3	0.5655	0.7016	0.5284
MICAN	**481.4**	0.5702	0.7040	0.5311
TMalign	479.7	**0.5710**	**0.7130**	**0.5329**
SSDT				
CLICK	360.2	0.5117	0.6815	0.5092
SPalignNS	345.9	0.4979	0.6530	0.4933
MICAN	**384.7**	**0.5304**	**0.7052**	**0.5207**
TMalign	339.1	0.4957	0.6541	0.4900
RIPC				
CLICK	951.2	0.6242	0.6039	0.5757
SPalignNS	945.0	0.6374	0.6119	0.5722
MICAN	**1083.6**	**0.6709**	**0.6399**	**0.5816**
TMalign	1050.1	0.6416	0.6114	0.5672

The result shows the qualities of the alignments after refinement. Initial alignments were generated by the external tools shown in the table. If the refined result of one method is better than that of the other, it can be assessed that the quality of the former initial alignment is better than the latter in terms of the scoring function used. The quality of the refined MICAN alignments consistently outperforms the others (SPalignNS, CLICK, and TMalign) except the HOMSTRAD set. Even in the HOMSTRAD set, the overall quality of the refined MICAN alignment is the second best with only small difference compared to the best result. Probably, the best alignments from HOMSTRAD set are largely in the form of sequential alignment and TMalign might have a strength on the sequential alignment. In any case, the success of the MICAN alignments in the refinement process does not mean that MICAN alignments themselves are high in terms of the scoring functions tested. Previous reports have shown that the score of MICAN alignment itself is lower in terms of SO-score [15]. We presume that MICAN alignments have lower SO-score because MICAN alignments disfavour much fragmented alignment blocks. Notwithstanding, the output of the refinements turns out to be best with MICAN alignments when minimum block size is set to 4. This implies that the MICAN alignment is more similar to the globally optimal alignment than the alignments generated by the other external tools.

### Comparison of scoring functions

The judgement for a better scoring function for alignment may depend on the purpose of the alignment. As discussed earlier, there is a trade off between the match length of the alignment and the RMSD value between the aligned part. However, if one alignment scoring function consistently generates alignments with longer match lengths and smaller RMSD values over another scoring function, it is reasonable to say that it is of better quality than the other. For the performance comparison of the four alignment scoring functions studied in this work (i.e., DALI-score, SO-score, SP-score, and TM-score,) ALIGN-CSA was used to generate optimal alignments for HOMSTRAD, SSDT, and RIPC sets. For each ALIGN-CSA run, we collected a total of 400 alignments using the minimum block size constraint of 4. However, the run was extended further if any locally refined alignments by external tools were of higher scores than those found by ALIGN-CSA. The results are summarized in Table 8, where the head-to-head comparison between two scoring functions is shown. The row-wise summations shown in the last column correspond to the total number of wins, and the column-wise summations shown in the bottom row correspond to the total number of losses. Therefore, a score function is considered to be better if the row-wise sum is large while the column-wise sum is small. We note that the total number of alignments considered in the three data set is 286 (64 + 199 + 23). Judging from the table, it is clear that the good scoring function are TM-score, SP-score, DALI-score, and SO-score in the order from the best to the worst according to the criteria. However, the difference between TM-score and SP-score is rather minor.

**Table 8:** The comparison of the efficiency of scoring functions. For each protein pairs of HOMSTRAD, SSDT, and RIPC sets, the best CSA solutions were compared. The numbers of better quality alignments of the row scoring function than the column scoring function are counted.

	DALI	SO	SP	TM	total wins
DALI	-	48	14	3	65
SO	11	-	0	7	18
SP	62	73	-	8	142
TM	22	117	4	-	143
total losses	95	238	18	17	

Although the longer match legnth and smaller RMSD can be a sure criterion for the better alignment in mathematical viewpoint, the biological relevance of the alignment still remains as a different issue. Both HOMSTRAD and RIPC set provide manually curated alignments and the alignments obtained in this research were compared with those reference alignments. Table 9 shows precision and recall of the alignments by ALIGN-CSA. Alignments with minimum block size 1 is generated by refining the alignments of ALIGN-CSA with minimum block size 4. Precision is TP/(TP + FP) and recall is TP/(TP + FN), where TP is true positive, FP is false positive and FN is false negative. In both test sets, DALI-score is absolutely superior in precision and recall for block size 4. When the minimum block size is 1, recall was the highest with TM-score in HOMSTRAD set. However, difference in recall between TM-score and DALI-score is minor. In RIPC set, DALI-score is also superior in both metrics. In conclusion, DALI-score function is good at reproducing the reference alignments. Generally, precision and recall are similar or lower with smaller block size. However, with TM-score, precision decrease and recall increases with the smaller block size. Out of all the combinations, DALI-score with block size 4 is selected as the best choice to obtain the best precision and recall.

**Table 9:** Average precision and recall of alignments by CSA-ALIGN. Global alignment was performed with minimum block size 4 (Block 4). Block 1 is the results of refinement of the alignments by CSA-ALIGN. At this refinement, block size restriction was released.

	Block 4		Block 1	
	Precision	Recall	Precision	Recall
HOMSTRAD				
DALI	0.909	0.845	0.902	0.845
SO	0.883	0.806	0.873	0.805
SP	0.893	0.777	0.876	0.774
TM	0.876	0.834	0.857	0.855
RIPC				
DALI	0.195	0.969	0.191	0.966
SO	0.115	0.733	0.116	0.725
SP	0.136	0.634	0.133	0.637
TM	0.106	0.774	0.103	0.789

**Fig 3:**
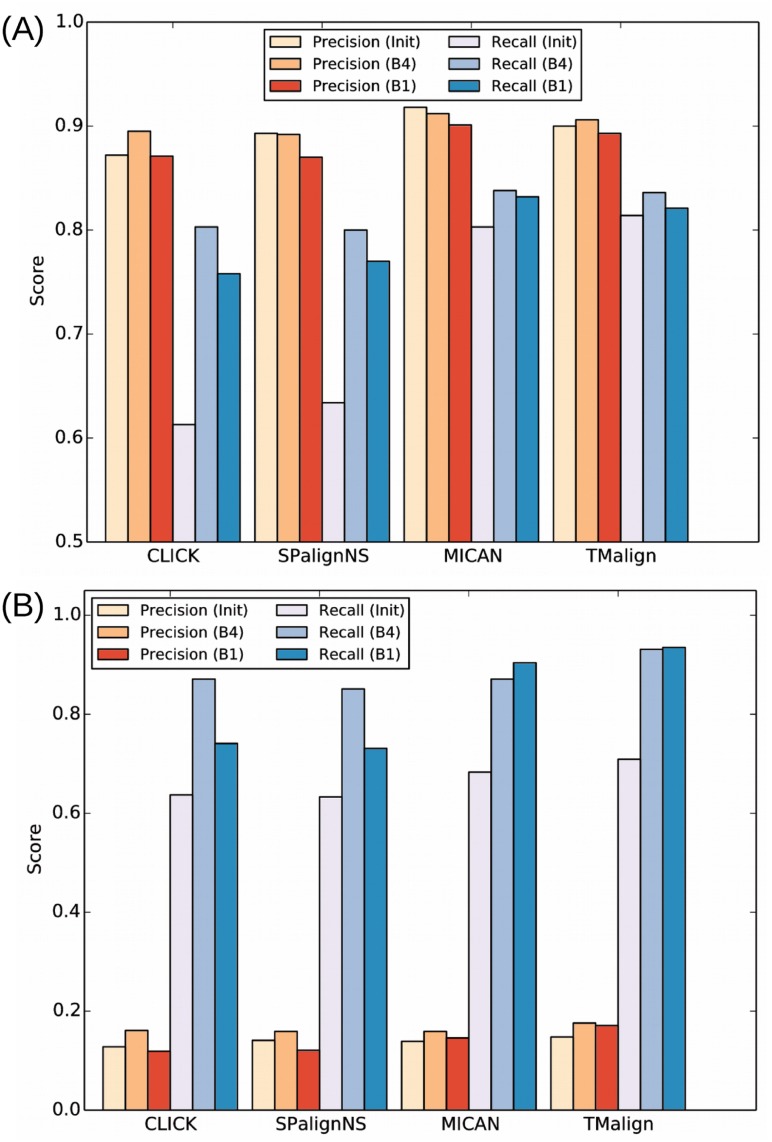
Effect of refinement on precision and recall. The plot shows the average precision and recall of the initial and refined alignments in two test sets (up: HOMSTRAD, down: RIPC). Two refinements with different minimum block sizes (1 and 4) were carried out.

**Fig 4:**
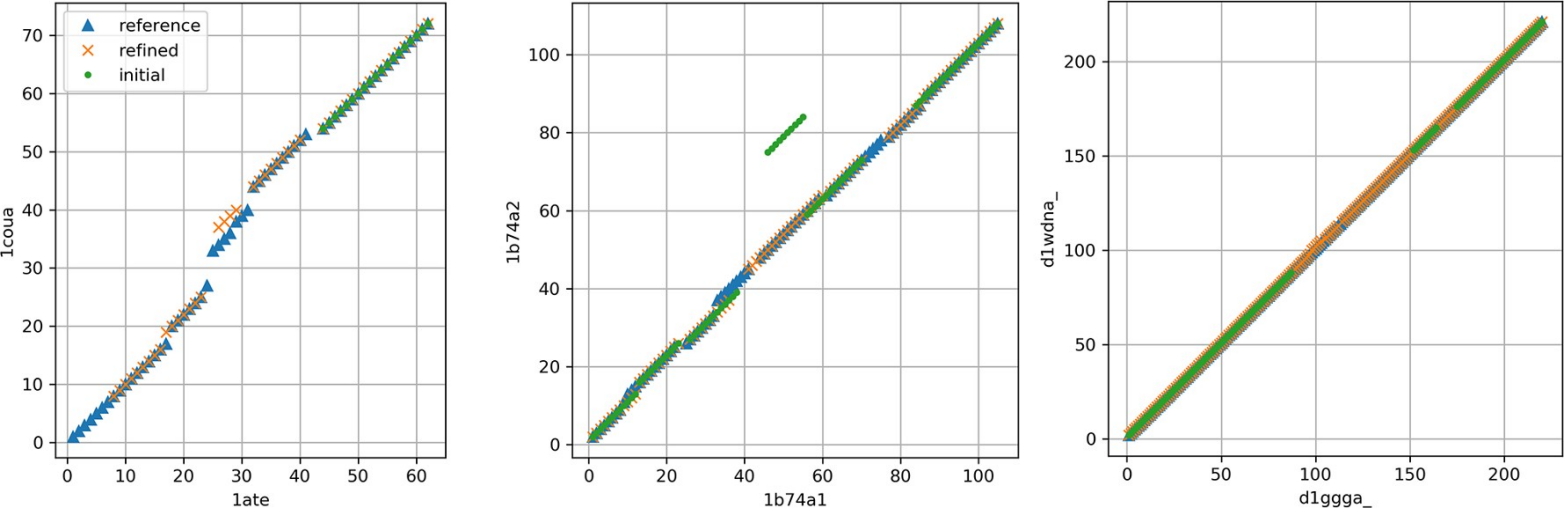
Three successful examples of refinement. The referential alignment (ref), initial MICAN alignment, and the refined alignment with the minimum block size 4 are shown. The refined alignments cover more of the referential alignments than the initial alignments.

Global alignment is useful in investigating scoring function and in the need of more optimized alignment. However, because of its longer running time (typically a few days of CPU time), it might be difficult to use the algorithm in some routine processes scanning database. Since the refinement process takes a few seconds to minutes, it can be a relatively quicker way to improve alignment score in such cases. Fig 3 shows whether the refinement is useful in obtaining better quality alignment. Alignments obtained by external alignment tools were refined with DALI-score and minimum block size restrictions were either 1 or 4. It shows the change of the precision and recall after refinement. When minimum block sizes 1 and 4 are compared, in most cases, alignments with minimum block size 4 are superior in terms of both precision and recall. In some cases, however, a minor gain in recall with minimum block size 1 is observed. Precision of alignments by SPalignNS and MICAN decrease after refinement in HOMSTRAD set. Even in these cases, DALI-score refinement with block size 4 enhances alignment quality by improving recall by large amount at little loss of precision. Some successful examples show that refined alignments cover more of the referential alignments (Fig 4). Therefore, refinement with DALI-score function with minimum block size 4 can be useful in improving the quality of a given alignment. However, we see that it will be necessary to investigate on a new scoring function to improve both the precision and recall consistently in the future.

## Conclusion

Topology-independent similarity between two protein structures can be detected only by non-sequential structure alignment. The structure alignment is usually carried out by optimizing a scoring function but obtaining the globally optimal solution is not trivial. Obviously, for proper evaluation of various scoring functions, a reasonably-efficient global search method is required. In this study, we proposed the ALIGN-CSA method capable of finding the global or near global alignment. Depending on the minimum block size and whether to allow the reverse alignment, the difficulty in finding the globally optimal alignment solutions varies. When the minimum block size is set to 4, the method shows quite robust results in spite of the stochastic nature of the method. The alignment found by ALIGN-CSA is expected to be (or close to) its global solution, and it can be used to benchmark the efficiency of various objective scoring functions. The qualities defined by match length and RMSD of the alignments obtained by four scoring functions showed that TM-score are the most effective followed by SP-score, DALI-score and SO-score, while the difference between the first two is marginal. The qualities defined by biological relevance of the alignments showed that DALI-score is apparently superior to the other score functions.

We proposed a refinement protocol based on a modified DGS algorithm to improve a given alignment in terms of any scoring function. Not only it improves the objective scoring function, but also it is possible to adjust the minimum block size or whether to allow the reverse alignment. Therefore, the protocol can be useful in removing unwanted much fragmented and/or reverse alignments blocks. The quality of the input alignment for the refinement protocol is critical since the refinement protocol performs only the local search. We compared the alignments obtained by SPalignNS, CLICK, TMalign, and MICAN and identified the alignment method that produces the best results after refinement. MICAN alignments improved the most by refinement. When the alignment is refined with DALI-score, Generally, the refined alignments showed higher precision and recall. In the refinement, minimum block size 4 showed higher quality than minimum block size 1.

Till now, many structure alignment studies have been reported, but due to the notorious difficulty to obtain globally optimal alignment solutions, all the existing methods were focused on generating “fast” and reasonably good alignment solutions. The current study distinguish itself as the first attempt to generate rigorous alignment solutions by investing significantly more computational resources compared to existing methods. Due to the slow running time, the proposed global search method, ALIGN-CSA, may not be suitable for all-to-all alignment of a large database. However, ALIGN-CSA can be useful to evaluate alignment scoring functions as performed in this work. In addition, ALIGN-CSA can be a viable option when more accurate alignments are required as in the study of protein structure prediction. As demonstrated on the 286 alignment problems of difficult cases (from HOMSTRAD, SSDT and RIPC), ALIGN-CSA generated significantly improved alignment results using various scoring functions. As an alternative means, a rather quick refinement method is proposed. The method can improve the quality or the score of a given alignment typically generated by existing alignment tools. We also note that it is straightforward to apply the methods proposed in this work to other macromolecules such as RNA, DNA and carbohydrates and any objective scoring functions not considered in this study. All programs are available from http://lee.kias.re.kr and minimal data are in S2 File.

## Supporting information

S1 File Supporting document. (PDF)

S2 File Supporting data. (GZ)
