# Supplementary material for: Non-sequential protein structure alignment by conformational space annealing and local refinement
Source: PLoS One. 2019 Jan 30;14(1):e0210177. doi: 10.1371/journal.pone.0210177 (PMC6353097; doi:10.1371/journal.pone.0210177)
Supplement: S1 File — (PDF) [file pone.0210177.s001.pdf]

Representation of Alignment

An alignment between two given protein structures A and B can be represented by a set of aligned residue pairs, $\{(a_i, b_i) : a_i$ and b_i are respectively residue indices of protein A and protein B forming an i-th aligned pair$\}$. First, we consider an alignment represented with respect to protein A. That is, below all the serial residue indices of protein A, corresponding aligned residue indices of protein B are placed (see Figure A). Under unaligned residue indices of protein A, gaps (denoted by -) are placed. In Figure Aa, two separate alignment solutions (s_1 and s_2) are shown with respect to protein A. Similarly, s_1 and s_2 can be represented with respect to protein B (see Figure Ab).

(a) Two alignment solutions s_1 and s_2 are represented by protein A's point of view.

Protein A's residue index	1	2	3	4	5	6	7	8	9	10	11
Protein B's residue index of s_1	-	1	2	3	4	-	9	10	11	12	-
Protein B's residue index of s_2	1	2	3	4	-	-	-	11	10	9	8
Index difference		1	1	1				1	1	3	

(b) The same two alignments of above are represented by protein B's point of view.

Protein B's residue index	1	2	3	4	5	6	7	8	9	10	11	12
Protein A's residue index of s_1	2	3	4	5	-	-	-	-	7	8	9	10
Protein A's residue index of s_2	1	2	3	4	-	-	-	11	10	9	8	-
Index difference	1	1	1	1					3	1	1	

Figure A: A schematic diagram to represent two alignments s_1 and s_2 and the distance between them. An alignment can be represented in two ways, (a) wrt. protein A and (b) wrt. protein B. To calculate the distance between s_1 and s_2, for each representation, the index difference between two aligned residues aligned to the identical partner is shown in the last row. The sum of these differences is defined as the distance between s_1 and s_2. In the example shown here, the distance between s_1 and s_2 is 8+9=17.

Definition of distance between two alignments

The distance between two given alignments is defined as the sum of the solution difference calculated in two ways as shown in Figure A. To measure the distance between s_1 and s_2, first, we consider all residue indices of protein A that are aligned to B in both alignments. The index difference of a protein B's residue aligned to the identical residue of protein A is calculated, as shown in the last row of Figure Aa. Similarly, the index difference of a protein A's residue aligned to the identical residue of protein B is calculated (see the last row of Figure Ab). The difference is only calculated when a residue is aligned. The sum of these differences is defined as the distance between s_1 and s_2.

Local optimizer

For local optimization of a given alignment, we used the quenching approach by trying various local perturbation moves and accepting only score-improving ones [2, 1].

We used three-step optimization as follows. In the first step, we used point extension, point deletion, and block extension (Figure B) in a random order. For point extension, an aligned pair is added to one end of an existing aligned block. For point deletion, an aligned pair is deleted as long as it does not violate the minimum block-size constraint. We note that a point deletion can split an aligned block into two as long as it does not violate the minimum block-size constraint. Block extension is similar to the point extension except that 2 or 3 contiguous residue pairs are extended simultaneously. When no improvement was observed after a complete round of all possible moves, we proceeded to the next step.

In the second step of quenching, block rolling was used by trying to shift all residues of one protein of an aligned block collectively. Four directions of block rolling are possible. For example, if residues (10, 11, 12, 13) of one

	-	6	7	8	9	-	-	-	-	-	···
point extension	**5**	6	7	8	9	-	-	-	-	-	···
point deletion	5	6	7	8	**-**	-	-	-	-	-	···
block extension	5	6	7	8	**9**	**10**	-	-	-	-	···
block rolling	-	**5**	**6**	**7**	**8**	**9**	**10**	-	-	-	···
block rolling	-	**4**	**5**	**6**	**7**	**8**	**9**	-	-	-	···
block deletion	-	4	5	6	7	**-**	**-**	-	-	-	···
block addition	-	4	5	6	7	-	**8**	**9**	**10**	**11**	···

Figure B: Various moves used to perturb a given solution. The denoted move is applied to the solution shown one line above, and the change of the move is shown in bold faces.

protein are aligned to residues (22, 23, 24, 25) of the other protein forming a block, the first ones can be shifted to either (9, 10, 11, 12) or (11, 12, 13, 14). Similarly, the second ones can be shifted to either (21, 22, 23, 24) or (23, 24, 25, 26). The maximum shift size of the block rolling was set to 8. When a move was accepted, the alignment was sent back to the first step described in the previous paragraph. Otherwise, we proceeded to the final step.

The final step performed block deletion followed by block addition. Block deletion considers deleting up to 7 contiguously aligned residue pairs as long as it does not violate the minimum block-size constraint. After a successful block deletion, the alignment was further tried with block addition, after which the alignment was sent back to the first step. Block addition adds a block of minimum-size contiguous residue pairs (Figure B). In the block addition, RMSD between two proteins was measured using the current alignment, and only those blocks in which all distances of the Cα pairs are within 220% of the RMSD value are tried. The block addition was tried up to 10,000 times. The quenching stopped when no improvement was observed.

Generation of a daughter solution

We consider two parent alignments p_1 and p_2, where the alignment is represented as a set of aligned residue pairs. Initially, the daughter alignment d is set as $d = p_1 \cap p_2$. In d, all aligned blocks are examined to satisfy the minimum block size constraint, and all under-sized blocks are extended using remaining residue pairs in $R = p_1 \cup p_2 - d$. To prevent potential double occupancy of a residue in d, whenever a residue pair t is moved from R to d, we remove residue pairs in R that contain a residue index of t, and the remaining set is denoted as R'.

We select a residue pair in R', t, in a random fashion. If the addition of t to d cannot satisfy the minimum block size constraint even after any follow-up operations, t is discarded. Otherwise, t is either discarded or moved to d with the equal probability. If necessary, additional residue pairs in R' are moved together with t to d, to satisfy the minimum block size constraint. This procedure is repeated until R' becomes a null set. If d is identical to one of its two parent alignments, it is discarded and the whole procedure is repeated up to 10 times.

CSA Procedure

With the three CSA ingredients described above, the overall procedure of CSA is carried out as follows. To start the first round of CSA, 50 alignment solutions are randomly generated. Specifically, 4-8 residue segments from one protein are paired by the same size segments from the other protein to form blocks of aligned pairs (forwardly or reversely). The total number of aligned residue pairs are set between 40 to 80 % of the smaller protein. All of the thus-generated alignments are subject to the local optimizer to constitute the first population of CSA, called *first bank*. We make a copy of *first bank* and call it *bank*. The solutions in *bank* are updated by better ones found during the

course of optimization, while *first bank* is kept unchanged. The initial value of D_{cut} is set to one half of the average distance among *first bank* solutions.

A total of 30 solutions are selected from *bank* as seeds. For each seed, 30 daughter solutions are generated by performing multiple crossover procedures as described above. Nine daughter solutions are generated by crossover between the seed and a randomly selected *bank* solution. In the early stage of CSA, represented by the number of unused seeds being greater than 47, three of the nine crossover operations are performed between two randomly selected *first bank* solutions. Three daughter solutions are generated by crossover between the seed and a randomly selected *first bank* solution.

Another eighteen daughter solutions are generated by crossover between two preprocessed alignments starting from the seed and a randomly selected *bank* solution. The preprocessing includes up to three operations of block rolling and up to ten combined operations of point extension and point deletion, in a random fashion. The sequence of block rolling and combined operation is also set in a random fashion. For each block rolling, the shift size is set to 1, and the operation is applied to a set of randomly selected blocks. The fraction of blocks for block rolling is also set in a random fashion. A combined operation of point extension and point deletion alternates between multiple point extensions and multiple point deletions, thus performing a fraction of all possible moves. The fraction is set in a random fashion.

These, 900 generated daughter alignments (30(seed)$\times$30(crossover)) are locally optimized as described in section "Local optimizer". Using each locally optimized daughter solution α, *bank* is updated as follows. If the score of α is worse than the worst score of bank, α is discarded. Otherwise, The closest alignment a in *bank* is identified with the distance of $D(\alpha, a)$. If $D(\alpha, a) \leq D_{\text{cut}}$, α replaces a if α is better than a. If $D(\alpha, a) > D_{\text{cut}}$, α replaces the worst solution in *bank*. We carry out this operation for all 900 daughter alignments to complete the update procedure. With thus updated *bank*, 30 new seed solutions are selected again from *bank* considering only those not used yet as seeds.

The entire process of generating daughter solutions, local optimizations and the updating of *bank*, is iterated until the number of unused seeds in *bank* becomes less than 10, signifying one complete round of CSA iteration. At each iteration step, D_{cut} is reduced with the ratio of 0.8513 so that D_{cut} reaches 20% of the initial value after 10 iterations. After D_{cut} reaches this value, it is kept constant till the end of the current round. At the end of a round of iterations, all solutions in *bank* are reset as "unused status" and another round of iterations starts. A total of three rounds of iterations are carried out.

Another 50 randomly generated and subsequently optimized solutions are added to both *bank* and *first bank* increasing the sizes of *bank* and *first bank* to 100. We repeat the whole three round procedure of aforementioned iterations. The value of D_{cut} is reset to one half of the average distance among solutions in *first bank*. In the first round of CSA iterations, the 50 newly added solutions in *bank* evolves in the same way as described above. But, seeds are taken only from the newly added 50 solutions and crossover is performed only between these newly added solutions. These two restrictions are released after the first round. The whole CSA procedure is completed after two additional rounds are carried out considering all 100 solutions. One may consider repeatedly adding additional 50 solutions to *bank* for a more thorough optimization at the expense of more computational resources.

References

[1] Seung-Yeon Kim, Sung Jong Lee, and Jooyoung Lee. Ground-state energy and energy landscape of the sherrington-kirkpatrick spin glass. *Physical Review B*, 76(18):184412, 2007.

[2] Juyong Lee, Steven P Gross, and Jooyoung Lee. Modularity optimization by conformational space annealing. *Physical Review E*, 85(5):056702, 2012.

ALIGN-CSA		Ref	
1lyzA	2lzmA	1lyzA	2lzmA
25-36	1-12	25-36	1-12
39-54	13-28	39-54	13-28
56-61	29-34	56-61	29-34
65-72	43-36	65-72	43-36
76-80	50-46	76-80	50-46
87-101	59-73	87-101	59-73
102-105	77-74	102-105	77-74
106-109	103-106	106-109	103-106
110-113	102-99	110-118	102-94
115-119	98-94	119-125	157-163
121-127	158-164		

(a) 1lyzA/2lzmA

ALIGN-CSA		Ref	
1colA	1sdhA	1colA	1sdhA
61-69	1-9	58-67	1-10
72-75	10-13	69-74	11-16
76-89	15-28	79-90	19-30
90-109	32-51	91-109	33-51
112-128	68-84	112-128	68-84
129-142	90-103	129-142	90-103
145-148	104-107	145-148	104-107
151-165	108-122	151-165	108-122
166-186	126-146	166-186	126-146

(b) 1colA/1sdhA

ALIGN-CSA		Ref	
1acxA	1cobB	1acxA	1cobB
1-8	13-20	1-8	13-20
9-13	5-1	15-25	26-36
15-25	26-36	26-36	38-48
26-36	38-48	39-42	59-62
39-42	59-62	43-46	80-77
43-46	80-77	48-64	82-99
48-64	82-99	66-69	104-101
66-69	104-101	73-78	69-64
73-78	69-64	80-83	107-110
80-83	107-110	87-95	112-120
87-95	112-120	96-106	139-149
96-106	139-149		

(c) 1acxA/1cobB

ALIGN-CSA		Ref	
1acxA	1tnfA	1acxA	1tnfA
1-8	98-91	1-8	98-91
12-15	127-130	13-16	127-130
16-24	84-76	17-24	83-76
28-36	149-157	28-36	149-157
41-44	11-14	41-44	11-14
48-51	15-18	48-51	15-18
53-56	138-141	55-63A	140-131
58-63A	137-131	64-67	48-51
64-67	49-52	68-71	41-38
68-71	41-38	75-79	6-10
75-79	6-10	85-94	53-62
86-94	54-62	97-106	117-126
96-106	116-126		

(d) 1acxA/1tnfA

ALIGN-CSA		Ref	
1acxA	1madH	1acxA	1madH
3-8	347-342	4-7	69-72
13-23	338-328	9-12	73-76
27-36	275-284	16-21	77-82
37-40	260-257	27-37	44-54
41-44	261-264	40-44	55-59
46-53	267-274	47-53	60-66
57-65	327-318	55-58	86-83
66-69	292-289	59-65	97-104
71-74	230-233	74-78	356-352
78-81	285-288	87-96	32-41
88-94	295-301	97-106	360-369
95-106	305-316		

(e) 1acxA/1madH

Table A: Original DALI alignments (Ref) vs the alignments by ALIGN-CSA with DALI-score

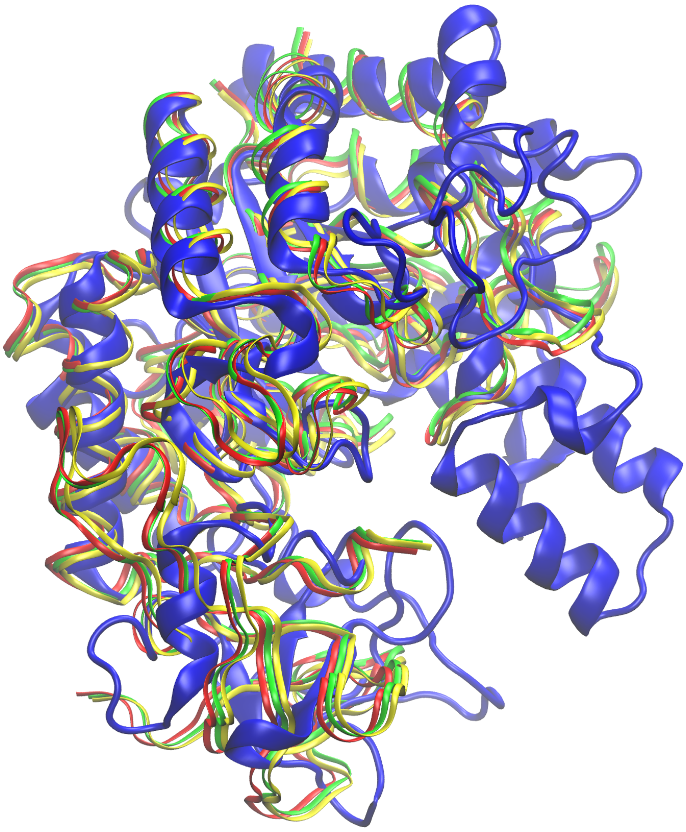

Figure C: Alingned molecules of 1uok and 1vjs from HOMSTRAD set. The blue molecule represents 1uok and other molecules are aligned 1vjs by reference alignment (red), ALIGN-CSA (green), and CLICK (yellow). The alignment of 1vjs in ALIGN-CSA is closer to the referential alignment in this case compared to the alignment of CLICK.

	Precision	Recall	Match length	DALI score	RMSD
ALIGN-CSA	0.754	0.880	323	3079.70	3.300
CLICK	0.699	0.640	263	2340.87	2.275
SPalignNS	0.742	0.749	282	2921.27	1.847
MICAN	0.777	0.866	307	2662.73	3.151
TMalign	0.750	0.870	317	2490.31	3.906

Table B: Alignment results of 1uok-1vjs by various alignment methods. Precision and recall are calculated based on the referential human-curated alignment. ALIGN-CSA used DALI-score as the score function. In this examplary case, the global optimization produces an alignment more similar to the referential alignment than CLICK, SPalignNS and TMalign. The precision and recall of the MICAN alignments probably because MICAN program is already trained to reproduce the referential alignments of HOMSTRAD set including this particular case. If the scoring function is finely tuned to be used in finding biologically relevant alignments, the usability of the global alignment may increase.

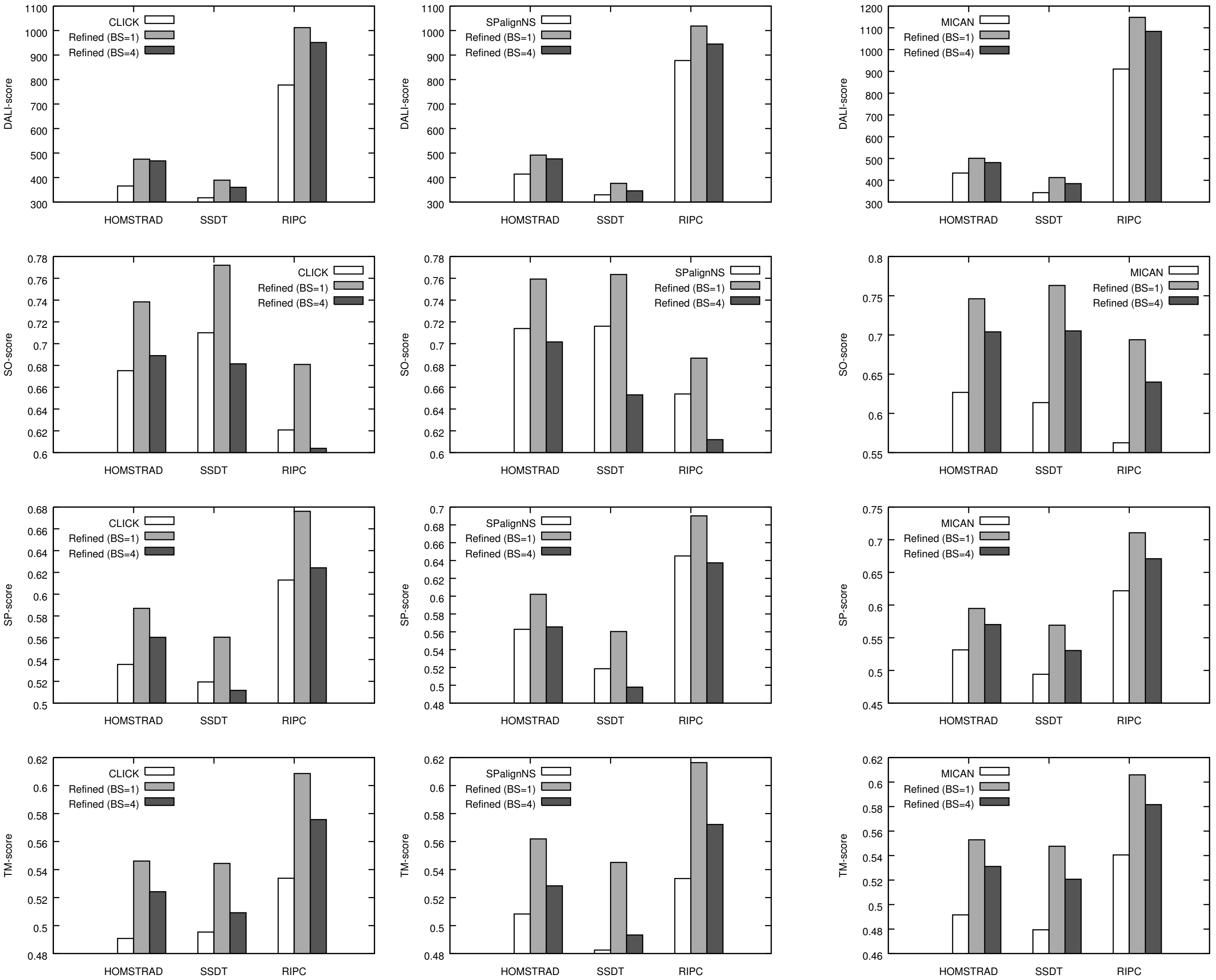

Figure D: Scores of refined alignments. The alignments of CLICK, SPalignNS, and MICAN were refined by optimizing DALI-score, SO-score, SP-score, and TM-score. The block size were restricted to either 1 (BS=1) or 4 (BS=4). The bars show the average scores of the alignments measured from the three test sets.
